# Protein Essential for Malarial Parasite to Reach and Infect Liver Cells

**DOI:** 10.1371/journal.pbio.0020032

**Published:** 2004-01-20

Plasmodium, the microscopic parasite that causes malaria, passes through two hosts, two reproductive modes, four habitats, and over half-a-dozen distinct developmental stages in one lifecycle. When a Plasmodium-infected mosquito bites a human, it injects the parasite—sequestered in the mosquito's salivary glands in its sporozoite stage—into the victim's bloodstream. Within hours, the sporozoites invade the liver—a critical stage for establishing infection—and spend the next few weeks asexually dividing inside liver cells, eventually releasing thousands of merozoites into the bloodstream. Merozoites quickly invade red blood cells and begin a second round of asexual proliferation. The infected cells rupture and die, releasing more parasites and toxins. The toxins cause malaria's characteristic fever and chills, and the liberated merozoites initiate another cycle of red blood cell attacks.

An unresolved question has been how the circulating sporozoites reach the liver cells in the first place, since liver cells are separated from the bloodstream by a layer of endothelial and Kupffer cells, which form the walls of the liver capillaries. (Kupffer cells project into the bloodstream and remove contaminants.) Having identified a protein required for sporozoite migration through the capillary lining, Tomoko Ishino, Masao Yuda, and their colleagues at Mie University School of Medicine in Japan may have found an answer.

Only four of the roughly 150 vertebrate-infecting Plasmodium species affect humans. P. falciparum, the most pathogenic of the human-infecting species, is closely related to avian and rodent species. One rodent species,—P. berghei—shares fundamental aspects of structure, physiology, and lifecycle with P. falciparum and so serves as a model for the human parasites. Since sporozoites must infect mosquito salivary glands before they can infect the mammalian liver, Yuda's team searched for sporozoite genes that are predicted to encode secretory or membrane proteins and are expressed only in mosquito salivary glands. Their search revealed a coding region conserved in several species of Plasmodium.

Tracing the gene's activity through the parasite's life cycle, Yuda's team confirmed that it was expressed only in sporozoites in the mosquito salivary gland—not in the mosquito midgut, where sporozoites are produced after mosquitoes feed on the blood of an infected person. The corresponding protein was localized to micronemes, specialized secretory organelles found at the front end of malaria parasites. Because micronemes are known to play a central role in Plasmodium motility and invasion, the researchers predicted this protein would also be important in migrating to or invading liver cells. They named the protein SPECT, for sporozoite microneme protein essential for cell traversal.

Yuda's team tested SPECT's function by generating *spect*-disrupted mutants and observing how the altered parasites affected their hosts. spect disruption did not affect parasite proliferation in rat red blood cells or interfere with parasite development in the mosquito midgut or salivary glands, but it did have an effect on the parasite's ability to infect the liver. Rats injected with *spect*-disrupted parasites had significantly lower levels of liver infection than rats injected with nonmutant parasites. Since it was unclear whether the *spect*-disrupted mutants lost their infectivity or simply could not pass through the cell layer, the researchers inoculated human liver cells with the mutants and found that they infected the cells normally.

Yuda's team also tested SPECT's impact on sporozoite cell-passage ability; if the mutants couldn't reach the liver cells, they couldn't infect them. *spect*-disrupted parasites completely lost their ability to pass through cells. Since traversal of the cellular barrier between liver cells and the circulatory system is a crucial step in malarial infection, the authors conclude, SPECT and other proteins involved in shuttling sporozoites into liver cells could be effective targets for malaria treatment and prevention.

**Figure:**
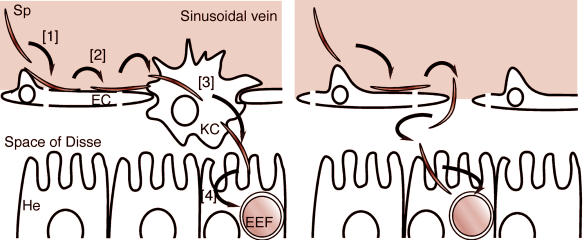
Sporozoite migration to hepatocytes

